# Reconfigurable quantum photonic circuits based on quantum dots

**DOI:** 10.1515/nanoph-2024-0044

**Published:** 2024-05-09

**Authors:** Adam McCaw, Jacob Ewaniuk, Bhavin J. Shastri, Nir Rotenberg

**Affiliations:** Centre for Nanophotonics, Department of Physics, Engineering Physics & Astronomy, Queen’s University, 64 Bader Lane, K7L 3N6, Kingston, Ontario, Canada; Vector Institute, M5G 1M1, Toronto, Ontario, Canada

**Keywords:** photonic integrated circuits, solid-state quantum emitters, chiral quantum optics, quantum information processing, programmable, phase shifters

## Abstract

Quantum photonic integrated circuits, composed of linear-optical elements, offer an efficient way for encoding and processing quantum information on-chip. At their core, these circuits rely on reconfigurable phase shifters, typically constructed from classical components such as thermo- or electro-optical materials, while quantum solid-state emitters such as quantum dots are limited to acting as single-photon sources. Here, we demonstrate the potential of quantum dots as reconfigurable phase shifters. We use numerical models based on established literature parameters to show that circuits utilizing these emitters enable high-fidelity operation and are scalable. Despite the inherent imperfections associated with quantum dots, such as imperfect coupling, dephasing, or spectral diffusion, we show that circuits based on these emitters may be optimized such that these do not significantly impact the unitary infidelity. Specifically, they do not increase the infidelity by more than 0.001 in circuits with up to 10 modes, compared to those affected only by standard nanophotonic losses and routing errors. For example, we achieve fidelities of 0.9998 in quantum-dot-based circuits enacting controlled-phase and – not gates without any redundancies. These findings demonstrate the feasibility of quantum emitter-driven quantum information processing and pave the way for cryogenically-compatible, fast, and low-loss reconfigurable quantum photonic circuits.

## 1 Introduction

Reconfigurable quantum photonic integrated circuits (qPICs) are versatile tools capable of simulating molecular dynamics [1], executing quantum logic [2], and generating multidimensional entanglement [3]–[7]. They utilize quantum properties such as entanglement and indistinguishability for information processing, which is unachievable through classical means. This capability is crucial for developing emerging quantum communication and computation technologies.

**Figure 1:**
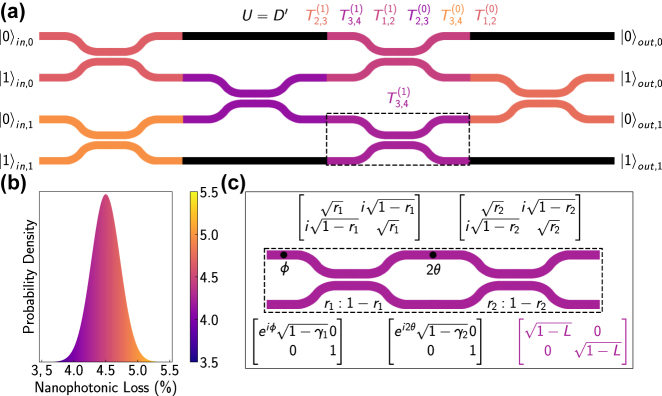
Scalable reconfigurable MZI meshes. (a) An exemplary 4-mode MZI mesh colored to represent nanophotonic losses in each interferometer. (b) Normal distribution for nanophotonic loss with a mean of 4.5 % and standard deviation that is 5 % of the mean. (c) A zoom-in on the basic component of the mesh (dashed region in a), a MZI with two phase shifters *ϕ* and 2*θ* and two imperfect beam splitters along with the imperfect transfer matrices for each component and for the overall nanophotonic loss.

To date, qPICs have predominantly operated at room temperature, harnessing the advancements of foundry photonics to create increasingly complex devices [8], [9]. As depicted in Figure 1, the core of these circuits is a mesh of Mach–Zehnder interferometers (MZIs) [10], where each MZI is comprised of two directional couplers and two phase shifters (see Figure 1(b)), typically thermo-optical in nature [11], [12]. That is, this mesh is fully classical, based on low-loss and highly accurate (albeit slow and hot) phase shifters. More recently, electro-optic [13], [14] and opto-mechanical [15], [16] phase shifters have become popular alternatives, however, they are limited to large footprints ( >100 μ m lengths) and MHz modulation speeds, respectively. Although any mesh, regardless of the type of phase shifter, is susceptible to unbalanced nanophotonic imperfections including losses and imperfect routing, high-fidelity operation is achievable with redundancy and optimization [17]–[22].

The quantum nature of qPICs stems from the individual photons that propagate through the circuits. Single photons are often generated by optical nonlinearities such as spontaneous parametric down-conversion, which is compatible with room-temperature chips but inherently probabilistic [23]–[25]. In contrast, single-photon sources based on solid-state quantum emitters, such as the quantum dots (QDs) we consider, can operate on-demand but require cryogenic temperatures [26]–[29]. Similarly, high-efficiency integrated single-photon detectors also necessitate a cryogenic environment [30]–[32]. As a result, both state-of-the-art sources and detectors cannot be heterogeneously integrated with qPICs and instead currently rely on lossy interconnects [33].

Here, we propose that QDs can be used not only as single-photon sources, but also as reconfigurable phase shifters for creating fast, cryogenically-compatible meshes. As photons scatter from QDs, and indeed all solid-state emitters, the imparted phase shift depends on the detuning between the photon and emitter transition frequency. For the case of QDs, the detuning can be modulated electrically [29], [34], optically [35], [36], or by using strain [37]. Our model of QD-based qPICs builds from this operating principle, yet additionally incorporates standard nanophotonic imperfections, such as losses and routing errors, along with QD-specific non-idealities, like imperfect interactions and both fast and slow noise processes. We use this model to evaluate the fidelity of both the resultant unitary operations and the desired output states. Our findings reveal that these QD-based meshes can be optimized to achieve remarkable scalability, with a unitary infidelity less than 0.001 for circuits up to 10 × 10 in dimension, using state-of-the-art QD parameters from the literature. We further consider QD-based controlled-phase and – not gates as examples, where we find that state-of-the-art circuits process logical states with fidelities of 0.9998. In sum, our results offer a roadmap to cryogenically-compatible, reconfigurable qPICs based on solid-state quantum emitters.

## 2 Quantum-emitter phase shifters

**Figure 2:**
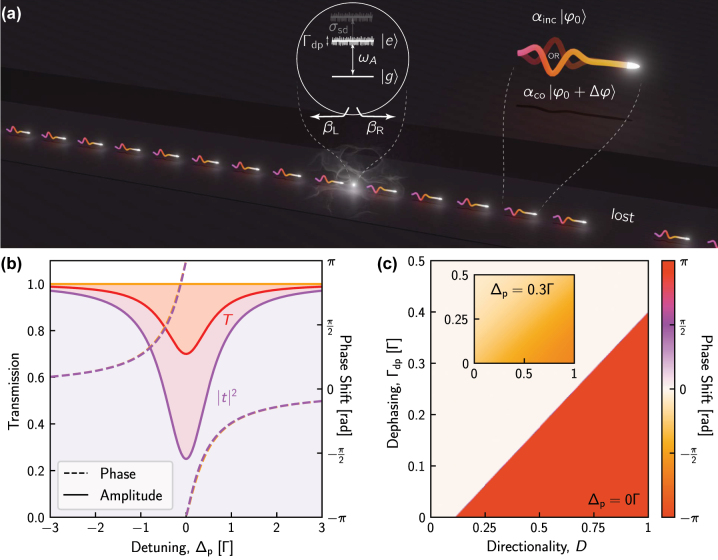
Waveguide-coupled chiral quantum dots (QDs). (a) A QD chirally-coupled to a waveguide with its forward direction to the right, *β*_R_ ≫ *β*_L_, imparts a phase shift Δ*φ* on coherently-scattered photons but not to those with which it interacts incoherently (due to fast dephasing). If *β*_R_ < 1, then some photons are either reflected or scattered out of the waveguide. (b) Transmission spectrum of the interaction shown in (a). In the ideal case (*β*_R_ = 1 and no noise, solid orange curve) all photons are transmitted, while if *β*_R_ = 0.9 and Γ_dp_ = 0.1Γ then the presence of the QD will be imprinted on the total transmission spectrum (solid red curve) and the coherent transmission spectrum (solid purple curve). The associated phase shift applied to the coherently-scattered photons in both the ideal (orange) and non-ideal (purple) scenarios is shown in the dashed curves (right axis), which are nearly identical such that they almost completely overlap. (c) On-resonance phase shift as a function of directionality and dephasing rate (inset: similar map for a photon-emitter detuning of Δ_p_ = 0.3Γ). The large region over which Δ*φ* = *π* demonstrates the robustness of QD-based phase shifters.

The scattering of photons from a quantum emitter embedded in a nanophotonic waveguide is a complex process [38] that may modulate the photons’ amplitude or phase [39], [40] or, when more than a single photon is present, induce complex correlations [35], [41]. The exact response depends on the properties of the emitter and the efficiency with which it couples to the various available modes, as sketched in Figure 2(a), yet in the most general case, an input single-photon state with phase *φ*_0_, ${\left|1\right\rangle }_{\text{in}}=\left|\varphi_{0}\right\rangle$ , will scatter into the mixed state,

(1) $${\left|1\right\rangle }_{\text{out}}=\left\{\begin{array}{c} \alpha_{\text{co}}\left|\varphi_{0}+\Delta \varphi \right\rangle \\ \alpha_{\text{inc}}\left|\varphi_{0}\right\rangle \end{array}\right.,$$

where, in the presence of losses the sum of the probabilities to scatter coherently and incoherently, |*α*_co_|^2^ and |*α*_inc_|^2^, do not sum to unity.

The description of Eq. (1) only holds if the single-photon pulse is not reshaped during scattering, requiring that the pulse linewidth *σ*_p_ be much shorter than that of the emitter. More formally, we require that *σ*_p_ ≤ Γ/1000, ensuring the phase shifter response is linear. As shown in the Supplementary Information, this condition holds for any two-level system, including QDs.

In this regime, the single-photon transmission coefficient *t* and total transmission *T* are the same as that of a weak coherent beam (see Supplementary Information for derivation),

(2) $$t=1-\Gamma \beta_{\text{R}}\frac{\Gamma_{2}+i\Delta_{\text{p}}}{\Gamma_{2}^{2}+\Delta_{\text{p}}^{2}},$$

(3) $$T=1-2\Gamma \Gamma_{2}\frac{\beta_{\text{R}}\left(1-\beta_{\text{R}}\right)}{\left(\Gamma_{2}^{2}+\Delta_{\text{p}}^{2}\right)},$$

where *β*_R_ is the coupling efficiency for right-traveling photons (with a total coupling efficiency *β* = *β*_R_ + *β*_L_), Δ_p_ is the detuning between the photon and emitter-transition frequencies, Γ is the decay rate of the emitter, and Γ_2_ = Γ/2 + Γ_dp_ where Γ_dp_ is the pure dephasing rate (i.e. fast noise). We note that in the presence of dephasing, $T\neq {\left|t\right|}^{2}$ (c.f. Figure 2(b)), and that the emitter might also suffer from slow noise leading to spectral diffusion with a characteristic linewidth *σ*_sd_.

Eqs. (2) and (3) allow us to quantify the results of the scattering. The coefficients, *α*_co_ and *α*_inc_, are related to the transmission, as shown in Figure 2(b). In the ideal case, where *β*_R_ = 1 and Γ_dp_ = 0, the transmission is always unity (orange curve) meaning that *α*_co_ = 1 and *α*_inc_ = 0. Conversely, in the presence of losses and/or dephasing, the situation is more complex with ${\left|\alpha_{\text{co}}\right|}^{2}={\left|t\right|}^{2}$ and ${\left|\alpha_{\text{inc}}\right|}^{2}=T-{\left|t\right|}^{2}$ as shown by the purple and red curves.

The phase of the coherently-scattered photons is likewise calculated from $\Delta \varphi =\arg\left(t\right)$ , here shown in dashed curves in Figure 2(b) corresponding to the ideal and non-ideal scenarios. As can be seen, the imparted phase shift is nearly identical in both cases, spanning the full 2*π* and demonstrating the robustness of emitter-based phase shifters. Full, 2D maps of the induced phase shift on resonance, Δ_p_ = 0, and at Δ_p_ = 0.3Γ, are shown in Figure 2(c) and its inset, respectively, again demonstrating that a 2*π* phase change is possible even when the directionality is below *D* < 0.25 or the dephasing rate is above Γ_dp_ > 0.3Γ. However, this range also depends on emitter parameters such as *β*. Corresponding maps of ${\left|\alpha_{\text{co}}\right|}^{2}$ are presented in the Supplementary Information. Together, these enable us to pick an emitter detuning for each desired phase shift, and then calculate the scattered state (c.f. Eq. (1)).

## 3 QD-based qPICs

**Figure 3:**
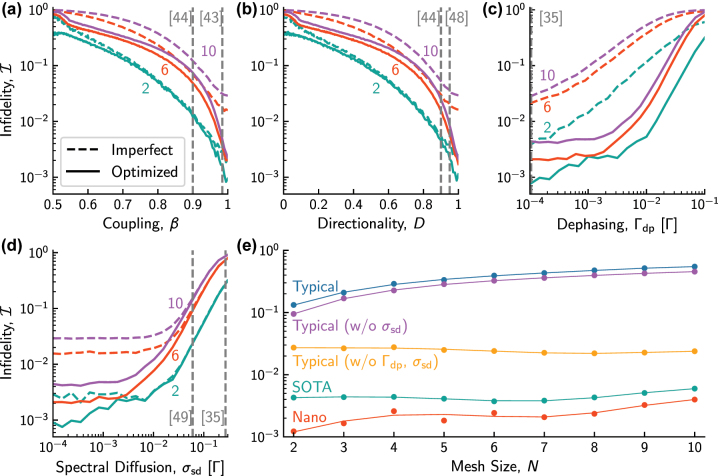
The effects of QD imperfections on circuit unitary infidelity. All plots have 4.5 % nanophotonic loss [8] and 4 % beam splitter error [21]. (a–d) With no other QD imperfections, we examine the effects of (a) coupling efficiency, *β*, (b) directionality, *D*, (c) pure dephasing rate, Γ_dp_, and (d) spectral diffusion, *σ*_sd_ on infidelity, showing both the imperfect (dashed) and optimized (solid) results. In all cases, state-of-the-art QD parameters from literature are indicated by vertical dashed lines. (e) The infidelity of QD-based qPICs as a function of mesh size ranging from *N* = 2 to 10 for circuits suffering only from nanophotonic imperfections (red), as well as those that additionally suffer from state-of-the-art QD imperfections (green) or typical QD imperfections (blue). See Table 1 for QD parameter values. Also shown are infidelities for circuits based on typical QDs but no noise (orange) or with only fast dephasing (purple), demonstrating that circuit errors are largely due to dephasing.

Having seen that quantum emitters such as QDs can serve as reconfigurable phase shifters, we quantify the performance of qPICs based on this technology. To do so, we first compare how well we can reproduce any unitary (i.e. operation) with our emitter-based qPICs relative to the ideal, summarizing the results in Figure 3. Here, we show the dependence of the mean circuit infidelity I (i.e. error) as a function of (a) *β*, (b) *D*, (c) Γ_dp_ and (d) *σ*_sd_, where, in all, I is limited by the nanophotonic errors as noted in the caption (see the Supplementary Information for details on how these were selected).

As an example, consider the *β*-dependence of I , shown in Figure 3(a). In dashed curves, we show the infidelity of the non-ideal unitary *U*_non_, constructed following the procedure of [10] using the set of phases $\left\{2\theta_{i},\phi_{i}\right\}$ (c.f. Figure 1(c)) calculated for the ideal circuit, but with imperfections subsequently added (see Supplementary Information for details). This corresponds to the offline training of a photonic circuit. For each coupling efficiency *β*, we compare 100 non-ideal unitaries *U*_non_ to the ideal *U* to calculate [10]

(4) $$I=1-{\left|\frac{tr\left(U^{†}U_{\text{non}}\right)}{\sqrt{Ntr\left(U_{\text{non}}^{†}U_{\text{non}}\right)}}\right|}^{2},$$

which we then average. In the figure, we show the cases for *N* = 2, 6, 10 mode circuits, where in all cases, we observe a monotonic increase from a baseline I due to the nanophotonic imperfections when *β* = 1, to near-unity error when the coupling efficiency is *β* = 0.5.

Encouragingly, we can optimize the performance of the emitter-based qPICs, following a fast and efficient routine that finds the optimal set $\left\{2\theta_{i},\phi_{i}\right\}$ at once [42]. This procedure results in an optimized unitary *U*_opt_ that, together with Eq. (4), enables us to again calculate I , which we show as solid curves in Figure 3. In all cases, we observe a significant reduction in errors due to the optimization, with this reduction particularly pronounced for larger circuit sizes and in near-optimal conditions. For example, in Figure 3(a), as *β* → 1 corresponding to state-of-the-art performance (dashed line) [43], we observe almost no increased infidelity as the circuit size increases. A similar dependence is observed as *D* and Γ_dp_ are scanned (Figure 3(b) and (c), respectively), while spectral diffusion only begins affecting the performance when *σ*_sd_ ≳ 0.01Γ (Figure 3(d)).

Overall, we summarize the circuit scaling in Figure 3(e), where we plot the raw and optimized infidelity as a function of circuit size, both using typical and state-of-the-art parameters (see Table 1). For typical values (blue curve), we see that the infidelity quickly approaches unity, yet by adding imperfections sequentially (purple and orange curves), we see that this is almost entirely caused by the residual dephasing. This is consistent with the optimized state-of-the-art qPIC performance (green curve), where I<0.006 is observed for all circuits simulated (up to *N* = 10), as several recent experiments based on QDs have measured Γ_dp_ ≈ 0 [35], [44]–[47].

**Table 1:** State-of-the-art and typical quantum dot parameters. Listed parameters include coupling, *β*, directionality, *D*, dephasing, Γ_dp_, spectral diffusion detuning standard deviation, *σ*_sd_. Though all parameters are taken from the literature, and are justifiable individually, it should be noted that they correspond to different types of waveguides. For more details on these parameters, including the reported uncertainties, see Section S3.1 of the Supplementary Information.

	Typical	Ref(s)	State-of-the-art	Ref(s)
*β*	0.90	[44]	0.9843	[43]
*D*	0.90	[44]	0.95	[48]
Γ_dp_	0.01Γ	[35]	0Γ	[35], [44]–[47]
*σ*_sd_	0.06Γ	[49]	0Γ	[50], [51]

## 4 Examples: CZ gate

**Figure 4:**
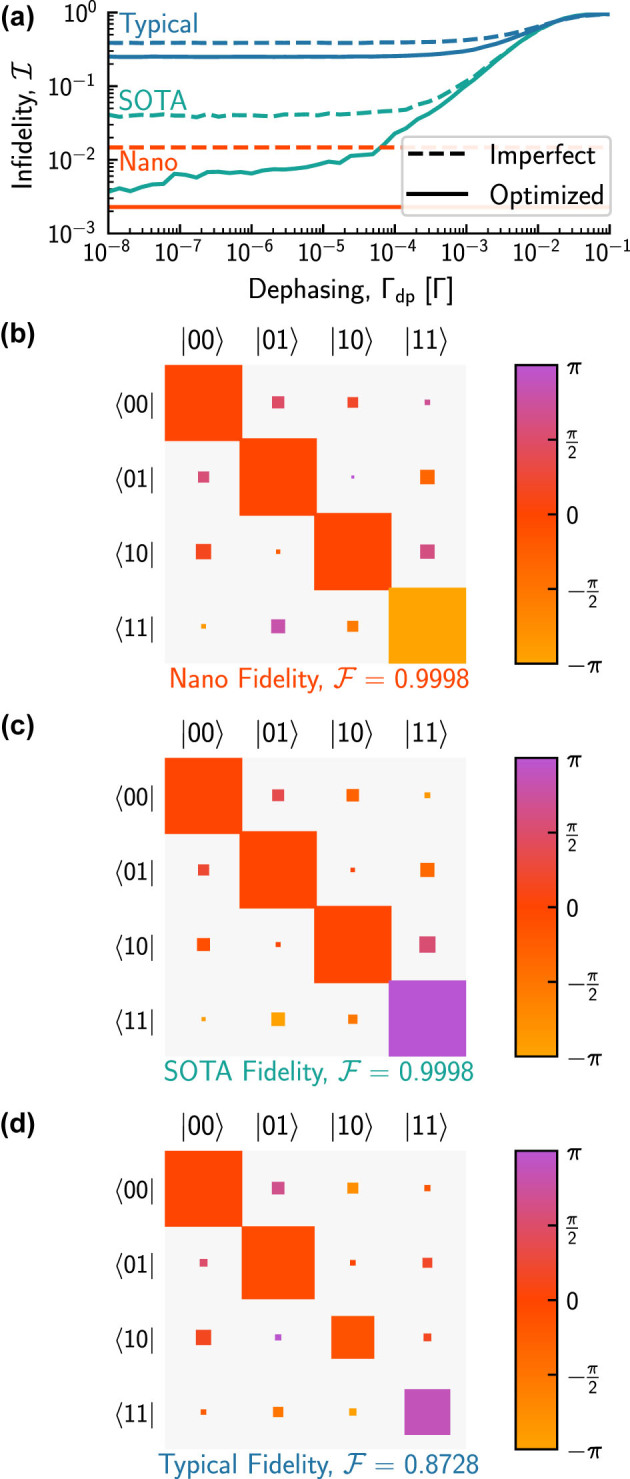
Unheralded CZ gate performance for nanophotonic, state-of-the-art, and typical imperfections (as previously explained in Figure 3 and Table 1). (a) Imperfect (dashed) and optimized (solid) unitary infidelities ( I , c.f. Eq. (4)) as a function of dephasing. All QD parameters other than Γ_dp_ are as listed in Table 1. Since circuits with only nanophotonic imperfections do not suffer from dephasing, their performance is flat in Γ_dp_. (b–d) Optimized nanophotonic, state-of-the-art, and typical post-selected 4 × 4 computational basis matrices for the unheralded CZ gate, with corresponding conditional output state fidelities listed below each matrix.

To demonstrate the possibilities of emitter-based qPICs, we consider the controlled-phase (CZ) gate, which can be used to generate entanglement [52], and, in the Supplementary Information, a similar realization of a controlled-not (CNOT) gate that enables universal quantum computation [53]. A linear-optical unheralded CZ gate can be realized on a 6 × 6 mesh [52], and in Figure 4(a) we plot the unitary infidelity I (Eq. (4)) calculated as a function of Γ_dp_ for circuits with only nanophotonic imperfections (red curve, no dephasing), the addition of typical QD imperfections (blue curve), and state-of-the-art QD imperfections (green curve), where for the latter two cases the dephasing varies along the horizontal axis (different from Table 1). For typical parameters, I>0.25 regardless of the dephasing rate, consistent with Figure 3. Interestingly, the optimized circuit based on typical QD parameters is nearly identical to that based on state-of-the-art when Γ_dp_ > 0.01Γ, meaning that in this regime, the effect of dephasing dominates all other emitter parameters. In contrast, the optimized curve for state-of-the-art parameters, including all QD imperfections (green), tends toward that for conventional phase shifters (red) in the limit that dephasing approaches its state-of-the-art value (Γ_dp_ ≈ 0). Additionally, while circuits with only nanophotonic imperfections achieve an optimized unitary infidelity of 0.0023, independent of Γ_dp_, it increases only to 0.0037 at Γ_dp_ = 10^−8^Γ, 0.0065 at Γ_dp_ = 10^−6^Γ, and remains less than 0.01 up to Γ_dp_ ∼ 10^−5^Γ.

More significantly, we consider the fidelity with which logical states are processed by the CZ gate (F) across Figure 4(b)–(d). Specifically, here we present the post-selected 4 × 4 matrices for the CZ gate in the computational basis for nanophotonic, state-of-the-art, and typical imperfections (as in Table 1). The state fidelity is the chance the CZ gate produces the correct output in the computational basis for any given input, given that the output is in the computational basis. It is shown below the corresponding matrix in each case (state fidelity calculation details are provided in the Supplementary Information). Although the performance of the typical circuit (Figure 4(d)) significantly differs from the nanophotonic-only (Figure 4(b)), as expected, the circuit based on state-of-the-art QD parameters (Figure 4(c)) achieves F=0.9998 , matching the performance of circuits constructed from traditional phase shifters. A similar figure and result is presented in the Supplementary Information for a CNOT gate. As demonstrated by these results, the state fidelity is typically better than the unitary fidelity, reinforcing the viability of QD-based qPICs.

## 5 Conclusions

Our research demonstrates that qPICs built with reconfigurable QD-phase shifters can perform comparable to those using classical phase shifters. Notably, we show that with state-of-the-art QD parameters, circuits can be scaled up to 10 modes without significant increases in unitary infidelity. This advancement allows QD-based qPICs to efficiently perform operations such as multi-qubit gates, as demonstrated in our study, and to simulate molecular dynamics [54] at cryogenic temperatures. In this respect, QD-based phase shifters join a select class that includes electro-optical [13], [14] and opto-mechanical [15], [16] shifters, but with a much smaller footprint, low operational energy and fast response times. As with current quantum photonic circuits, the performance of emitter-based systems could be further enhanced by incorporating redundancies, where extra MZIs and phase shifters provide better compensation for imperfections [17], [19]–[21].

We recognize that while QDs are the only quantum emitters currently integrated into photonic circuits, alternative technologies based on single organic molecules [55], [56] and defects in diamond [57], [58], silicon [59], or silicon carbide [60], [61] are rapidly maturing. In fact, even with QDs, not all state-of-the-art parameters have been demonstrated using a single chiral quantum photonic platform (c.f. Table 1). To date, however, high quality chiral quantum interfaces have been demonstrated with nanobeams [48], glide-plane waveguides [44] and topological photonics [62], [63]. Recent calculations suggest that, were the emitter to be placed at exactly the correct location within either photonic resonators [64] or waveguides [65], these could act as near-ideal chiral interfaces. This is particularly promising in light of recent developments demonstrating the ability to pre-select specific QDs and integrate them deterministically within a circuit [66]–[71].

Finally, we note that fabricating and managing circuits with numerous emitters remains a topic of inquiry. Recent experiments with QDs have successfully demonstrated the integration of deterministic QD-based single-photon sources with qPICs [72], [73], and independent control of multiple emitters within a circuit [74]–[76], respectively. These advancements open the door to larger-scale implementations where emitters would function both as sources and processing elements. Such circuits would be entirely cryogenic, and thus compatible with deterministic sources and detectors, with phase shifters whose operational speeds are determined by emitter lifetimes, potentially enabling GHz rate operation with mild enhancement [50].

## Supplementary Material

Supplementary Material Details
