# Supplementary material for: Reconfigurable quantum photonic circuits based on quantum dots
Source: Nanophotonics. 2024 May 9;13(16):2951–9. doi: 10.1515/nanoph-2024-0044 (PMC11245123; doi:10.1515/nanoph-2024-0044)
Supplement: Supplementary file 1 — Supplementary Material Details [file j_nanoph-2024-0044_suppl_001.pdf]

Supplementary Information

Adam McCaw, Jacob Ewaniuk, Bhavin J. Shastri, and Nir Rotenberg*

Reconfigurable quantum photonic circuits based on quantum dots

S1 Transmission and Phase Shift from a Chirally Coupled Quantum Dot

A quantum dot can be modeled as a two-level system (TLS) with a ground state $|g\rangle$ and an excited state $|e\rangle$, allowing their light-matter interaction to be modeled using the Jaynes-Cummings Hamiltonian [S1] as,

$$\hat{H}_S = -\hbar\Delta_P\hat{\sigma}_{eg}\hat{\sigma}_{ge} + \hbar\omega_P\hat{f}(r)^\dagger\hat{f}(r) - \hat{d}\cdot\hat{E}(r). \tag{S1}$$

The first term describes the dynamics of the emitter's TLS where $\hat{\sigma}_{ij} = |i\rangle\langle j|$, and $\Delta_P = \omega_P - \omega_A$ is the detuning between the driving light field frequency, ω_P, and the emitter's resonant frequency, ω_A. The second term, with the bosonic creation and annihilation operators $\hat{f}^\dagger$ and $\hat{f}$, describes the energy of the free field at the position r. The final term describes the light-matter interaction between the transition dipole of the emitter, $\hat{d} = d^*\hat{\sigma}_{eg} + d\hat{\sigma}_{ge}$, and the electric field, $\hat{E} = \hat{E}^+ + \hat{E}^-$. Using the rotating wave approximation and the Lindblad master equation [S2], we can determine the steady-state solution for the response of the TLS in terms of its reduced density matrix elements ρ_{ij} as,

$$\begin{aligned}\rho_{ee} &= \frac{2\Gamma_2\Omega_P^2}{\Gamma(\Gamma_2^2 + \Delta_P^2 + 4(\Gamma_2/\Gamma)\Omega_P^2)}\\ \rho_{ge} &= \frac{-\Omega_P(i\Gamma_2 + \Delta_p)}{\Gamma_2^2 + \Delta_P^2 + 4(\Gamma_2/\Gamma)\Omega_P^2},\end{aligned} \tag{S2}$$

where $\Gamma = \Gamma_{ee}$ is the rate of spontaneous emission of the TLS, also corresponding to the natural linewidth of the emitter in the frequency spectra. $\Gamma_2 = \Gamma/2 + \Gamma_{dp}$ where Γ_{dp} is the rate of dephasing in the system, and $\Omega_P = d\cdot E/\hbar$ is the Rabi frequency of the light-matter system.

The electric field operators for the light field can be written in terms of the Green's tensor $\overleftrightarrow{G}(r, r')$, the solution for the electric field operator of a point source, and the bosonic creation and annihilation operators [S3] as,

$$\begin{aligned}\hat{E}^+(r,\omega_P) &= i\omega_P^2\mu_0\sqrt{\frac{\hbar}{\pi}\epsilon_0}\int_{-\infty}^{\infty} dr'\sqrt{\epsilon_I(r',\omega_P)}\overleftrightarrow{G}(r,r')\cdot\hat{f}(r',\omega_P)\\ \hat{E}^-(r,\omega) &= -i\omega_P^2\mu_0\sqrt{\frac{\hbar}{\pi}\epsilon_0}\int_{-\infty}^{\infty} dr'\sqrt{\epsilon_I(r',\omega_P)}\overleftrightarrow{G}^*(r,r')\cdot\hat{f}^\dagger(r',\omega_P).\end{aligned} \tag{S3}$$

Equations S1 and S3 allow us to write the time evolution of the bosonic annihilation operator using the Heisenberg Equation of motion as,

$$\begin{aligned}\dot{\hat{f}}(r,\omega_P) &= \frac{i}{\hbar}\left[\hat{H},\hat{f}(r,\omega_P)\right]\\ &= -i\omega_P\hat{f}(r,\omega_P) + \omega_P^2\mu_0\sqrt{\frac{\hbar}{\pi}\epsilon_0}d(r_A)\sqrt{\epsilon_I(r,\omega_P)}\overleftrightarrow{G}^*(r_A,r,\omega_P)\hat{\sigma}_{ge},\end{aligned} \tag{S4}$$

where r_A is the position of the emitter in the waveguide. Formally integrating this equation from time t' to t results in [S4],

$$\hat{f}(r,\omega_P,t) = \hat{f}(r,\omega_P,t')\exp(-i\omega_P(t-t'))+ \omega_P^2\mu_0\sqrt{\frac{\hbar}{\pi}\epsilon_0}\int_0^t dt' d(r_A)\sqrt{\epsilon_I(r,\omega_P)}\overleftrightarrow{G}^*(r_A,r,\omega_P)\hat{\sigma}_{ge}(t')e^{-i\omega_P(t-t')}, \quad \text{(S5)}$$

where the first term describes a free excitation in the system that does not interact with the emitter, and the second term describes an interaction with the emitter either through a decay from excited to ground state or through a scattering event (virtual transition). Thus, we can write the electric field operator as,

$$\hat{E}^+ = \hat{E}_P^+ + \hat{E}_S^+, \quad \text{(S6)}$$

where $\hat{E}_P^+$ represents the incident field and $\hat{E}_S^+$ represents the scattered field. The transmission coefficient for the light in the system is written in terms of the expectation values of these fields as,

$$t = \frac{\langle \hat{E}^+ \rangle}{\langle \hat{E}_P^+ \rangle}, \quad \text{(S7)}$$

and the phase shift on the light from the interaction with the emitter is,

$$\phi = \arg(t). \quad \text{(S8)}$$

Equations S3 and S5, with the help of the Green's tensor identity [S3] and Kramer's Kronig relations [S5], allow the scattered field in terms of the incident field as,

$$\hat{E}_S^+(r,t) = \frac{1}{\hat{\Omega}_P} g(r,r_A,\omega_A)\hat{\sigma}_{ge}(t)\hat{E}_P^+(r_A,t), \quad \text{(S9)}$$

where we have defined the Rabi frequency operator as $\hat{\Omega}_P = d^* \cdot \hat{E}_P^+/\hbar$, where $\langle \hat{\Omega}_P \rangle = \Omega_P$, and we use the dipole-projected Green's function [S3],

$$g(r_i,r_j,\omega) = \frac{\mu_0\omega^2}{\hbar} d^*(r_i)\cdot G(r_i,r_j,\omega)\cdot d(r_j). \quad \text{(S10)}$$

For a chirally coupled quantum dot, the dipole projected Green's function becomes,

$$g(r,r_A,\omega) = i\Gamma(\Theta(r_A-r)\beta_L + \Theta(r-r_A)\beta_R)e^{ik_p|r-r_A|}, \quad \text{(S11)}$$

where Θ is the Heaviside function and we define the couplings for photons moving left β_L and for photons moving right β_R as,

$$\beta_L = \frac{\Gamma_L}{\Gamma} = \frac{\Gamma_L}{\Gamma_L+\Gamma_R+\Gamma_{Loss}}, \quad \text{(S12)}$$

$$\beta_R = \frac{\Gamma_R}{\Gamma} = \frac{\Gamma_R}{\Gamma_L+\Gamma_R+\Gamma_{Loss}}. \quad \text{(S13)}$$

In the low-power regime (weak coherent beam) where $\Omega_P \to 0$, the scattering event that imparts a phase shift will dominate over spontaneous emission, allowing the emitter to act as a phase shifter. From Equations S2 and S7 the transmission coefficient becomes,

$$t = 1 - \Gamma\beta_{\mathrm{R}}\frac{\Gamma_2 + i\Delta_{\mathrm{p}}}{\Gamma_2^2+\Delta_{\mathrm{p}}^2}, \quad \text{(S14)}$$

where we have defined the forward direction as right for simplicity. The phase shift can then be calculated with Equation S8. With the same assumptions, the observable transmission, T, can be written as,

$$T = \frac{\langle \hat{E}^-\hat{E}^+ \rangle}{\langle \hat{E}_P^-\hat{E}_P^+ \rangle}$$

$$T = 1 - 2\Gamma\Gamma_2\frac{\beta_{\mathrm{R}}(1-\beta_{\mathrm{R}})}{(\Gamma_2^2+\Delta_{\mathrm{p}}^2)}. \quad \text{(S15)}$$

S2 Ideal Phase Solution Using Unitary Decomposition

To solve the ideal phase shifts for a target unitary we follow the decomposition and recombination method proposed by Clements et al. [S6] where an ideal MZI is built up from the 2×2 transfer matrices of two $50:50$ directional couplers and two phase shifters (ϕ, 2θ) as,

$$\begin{aligned} MZI &= \frac{1}{\sqrt{2}}\begin{bmatrix} 1 & i \\ i & 1 \end{bmatrix}\begin{bmatrix} e^{i2\theta} & 0 \\ 0 & 1 \end{bmatrix}\frac{1}{\sqrt{2}}\begin{bmatrix} 1 & i \\ i & 1 \end{bmatrix}\begin{bmatrix} e^{i\phi} & 0 \\ 0 & 1 \end{bmatrix} \\ &= ie^{i\theta}\begin{bmatrix} e^{i\phi}sin\theta & cos\theta \\ e^{i\phi}cos\theta & -sin\theta \end{bmatrix}, \end{aligned} \tag{S16}$$

which can be expanded into the $N \times N$ matrix,

$$T^{(p)}_{m,n} = \begin{bmatrix} 1 & 0 & \dots & \dots & \dots & 0 \\ 0 & \ddots & \dots & \dots & \dots & \vdots \\ \vdots & \dots & ie^{i\theta}e^{i\phi}sin\theta & ie^{i\theta}cos\theta & \dots & \vdots \\ \vdots & \dots & ie^{i\theta}e^{i\phi}cos\theta & -ie^{i\theta}sin\theta & \dots & \vdots \\ \vdots & \dots & \dots & \dots & \ddots & 0 \\ 0 & \dots & \dots & \dots & 0 & 1 \end{bmatrix}, \tag{S17}$$

representing the pth MZI between modes m and n in the circuit. To solve for the ideal phases the Clements method [S6] then decomposes the unitary into a diagonal matrix D by applying MZIs/inverse MZIs using each of the $N(N-1)/2$ MZIs to nullify an off-diagonal entry. The result for a 4×4 unitary is,

$$\tilde{T}^{(1)}_{3,4}\tilde{T}^{(1)}_{2,3}UT^{(0)-1}_{1,2}T^{(0)-1}_{3,4}T^{(0)-1}_{2,3}T^{(1)-1}_{1,2} = D, \tag{S18}$$

where the indicates that these MZIs do not correspond to the hardware MZIs as they will be changed during the recombination steps. To determine the required phases for these MZIs, the algorithm is as follows; If a $T^{(p)-1}_{m,n}$ is being applied, the phases are chosen as,

$$\begin{aligned} \theta &= \frac{\pi}{2} - \arctan\left(\left|\frac{U_{Null}[x,m]}{U_{Null}[x,n]}\right|\right) \\ \phi &= \pi + \arg\left(\frac{U_{Null}[x,m]}{U_{Null}[x,n]}\right), \end{aligned} \tag{S19}$$

where U_{Null} corresponds to the partially decomposed matrix at the current decomposition step, and the index x is the row index of the element being nullified. Conversely, if a $T^{(p)}_{m,n}$ is being applied to the LHS, the phases are chosen as,

$$\begin{aligned} \theta &= \frac{\pi}{2} - \arctan\left(\left|\frac{U_{Null}[n,y]}{U_{Null}[m,y]}\right|\right) \\ \phi &= \pi + \arg\left(\frac{U_{Null}[n,y]}{U_{Null}[m,y]}\right), \end{aligned} \tag{S20}$$

where now the column index y corresponds to the column of the element being nullified.

For the recombination step, isolate for U in Equation S18, and shift the diagonal matrix to the left, replacing it and the $\tilde{T}^{(p)-1}_{m,n}$'s according to,

$$\tilde{T}^{(p)-1}_{m,n}D = D_1T^{(p)}_{m,n}, \tag{S21}$$

where D_1 is another diagonal matrix and $T^{(p)}_{m,n}$ is the MZI that corresponds to the hardware implementation. The new phases for $T^{(p)}_{m,n}$, following the Clements solution are,

$$\theta = \frac{\pi}{2} - \arctan\left(\left|\frac{M[n,m]}{M[n,n]}\right|\right)$$
$$\phi = \pi + \angle\frac{M[n,m]}{M[n,n]}, \tag{S22}$$

where $M = \tilde{T}^{-1}_{m,n,p}D$. From here, we determine the new diagonal matrix as,

$$D_1 = \tilde{T}^{(p)-1}_{m,n} D T^{(p)-1}_{m,n}. \tag{S23}$$

By repeating these steps until D is on the left side of the formula, we get, for a 4×4, an equation for the ideal unitary as,

$$U = D' T^{(1)}_{2,3} T^{(1)}_{3,4} T^{(1)}_{1,2} T^{(0)}_{2,3} T^{(0)}_{3,4} T^{(0)}_{1,2}, \tag{S24}$$

where we have relabelled the final diagonal matrix as D', which corresponds to global phase shifts applied at the end of the MZI mesh.

S3 Imperfect Transfer Matrix Generation

Imperfections are added to the transfer matrices at the MZI level, which combine to form an imperfect transfer matrix using Equation S24. The first imperfection we consider is nanophotonic loss, originating from waveguide propagation losses and beam splitter losses. For this, we use the common [S7–S10] assumption that loss is balanced across MZI paths, modeling the loss as,

$$Loss = \begin{bmatrix} \sqrt{1-L} & 0 \\ 0 & \sqrt{1-L} \end{bmatrix}. \tag{S25}$$

Beam splitter error on the directional couplers is also included, modeled using the standard variable beam splitter matrix,

$$BS = \begin{bmatrix} \sqrt{r} & i\sqrt{1-r} \\ i\sqrt{1-r} & \sqrt{r} \end{bmatrix}, \tag{S26}$$

where r is the reflectivity of the beam splitter and $r = 0.5$ in the ideal case.

Past experiments involving photonic integrated circuits have demonstrated typical MZI insertion losses ranging from 1.15% to 4.5% [S7]. These values all include the losses contributed by the two phase shifters in each MZI, though we include losses from the QD phase shifters in addition to these losses as discussed below. Regardless, since our study is platform-independent, it is best to make a conservative choice. Therefore, we take 4.5% nanophotonic loss per MZI. With regards to beam splitter error, we similarly make the conservative choice of 4%, as consistent with Ref. [S11], even though these may be as low as 0.23% [S12].

Quantum dot phase shift imperfections are factored into the model as well. Imperfect coupling, $\beta = \beta_L + \beta_R < 1$, and imperfect directionality, $D = (\beta_R - \beta_L)/(\beta_L + \beta_R) < 1$, are included through their effects on quantum dot loss, modeled as $\gamma = 1 - T$, where T is the observable transmission in the system calculated using Equation S15. This is included using the lossy phase shift matrix,

$$PS = \begin{bmatrix} \sqrt{1-\gamma}e^{\phi} & 0 \\ 0 & 1 \end{bmatrix}. \tag{S27}$$

Figure S1 depicts how the quantum dot loss γ is sampled for each phase shifter from a distribution with a central value $\gamma = 1 - T$ with a standard deviation of 5% of the central value to model

fluctuations between different quantum dots. Combining all these imperfections into the model, each MZI is calculated using the imperfect matrix,

$$MZI = \begin{bmatrix} \sqrt{r_2} & i\sqrt{1-r_2} \\ i\sqrt{1-r_2} & \sqrt{r_2} \end{bmatrix} \begin{bmatrix} \sqrt{1-\gamma_2}e^{i2\theta} & 0 \\ 0 & 1 \end{bmatrix} \begin{bmatrix} \sqrt{r_1} & i\sqrt{1-r_1} \\ i\sqrt{1-r_1} & \sqrt{r_1} \end{bmatrix} \begin{bmatrix} \sqrt{1-\gamma_1}e^{i\phi} & 0 \\ 0 & 1 \end{bmatrix} \begin{bmatrix} \sqrt{1-L} & 0 \\ 0 & \sqrt{1-L} \end{bmatrix}, \tag{S28}$$

which is then combined with all other imperfect MZIs to build an $N \times N$ imperfect transfer matrix. This is depicted as a circuit in Figure S1a, which shows how MZIs combine to form a 4×4 circuit, where Figure S1c shows the breakdown of each MZI component with its appropriate transfer matrix.

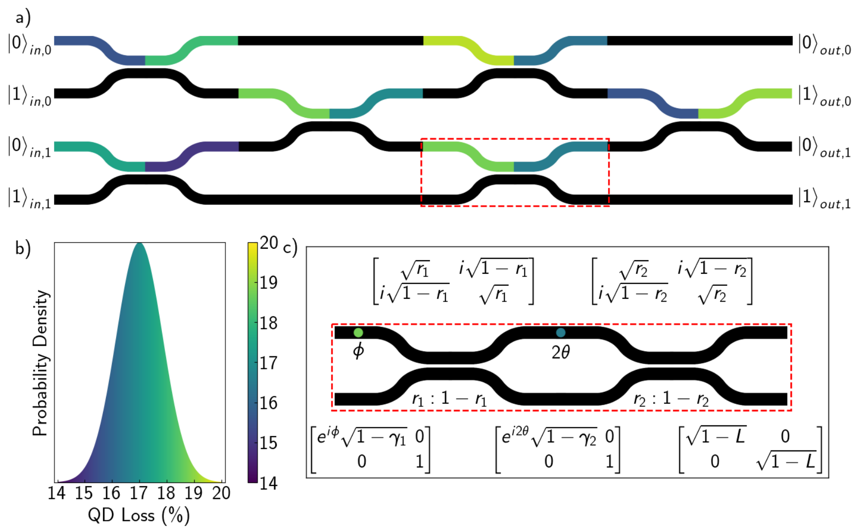

Fig. S1: Imperfect circuit transfer matrix generation with example quantum dot loss distribution. (a) 4×4 MZI mesh circuit depicting QD loss for each quantum dot in the MZIs. The color in the top-left branch of each MZI indicates the loss for the ϕ phase shifter, and the color in the top-right branch of each MZI indicates the loss for the 2θ phase shifter. If the path is black this indicates no QD loss in that region of the circuit. (b) Normal distribution for QD loss with a central value of 17% and a standard deviation of 5% of 17%. (c) MZI with transfer matrices for the two QD phase shifters with losses γ_1 and γ_2 as colored, the two beam splitters with reflectivities r_1 and r_2 and nanophotonic loss per MZI of L.

This imperfect transfer matrix does not include dephasing and spectral diffusion, which are included in QD imperfections. Dephasing provides a chance of having an incoherent interaction with an average phase shift of 0, calculated as $|\alpha_{\text{inc}}|^2 = (T - |t|^2)/T$. Consequently, the probability of a coherent interaction is $|\alpha_{\text{co}}|^2 = 1 - |\alpha_{\text{inc}}|^2$. Figure S2 depicts the coherence probability based on dephasing and directionality for detunings of $\Delta_P = 0\Gamma$ in Figure S2a and $\Delta_P = 0.3\Gamma$ in Figure S2. The larger the detuning, the higher the coherence probability, since there is less interaction with the quantum dot and thus less dependence on dephasing.

Dephasing is modeled using a Monte-Carlo simulation with 500 samples, where in each trial each phase shifter is sampled to be on/off based on its QD's incoherence probability, generating 500 imperfect unitaries on which we average the performance.

Spectral diffusion is also included in these Monte-Carlo simulations, where for each phase shifter we sample a shift in detuning, and consequently phase, from a normal distribution with a standard deviation of σ_{SD}, modeling the fluctuations from spectral diffusion in the system. Figure S3 depicts the spectral diffusion effects on detuning and phase shift for an example spectral diffusion of $\sigma_{\text{SD}} = 0.06\Gamma$.

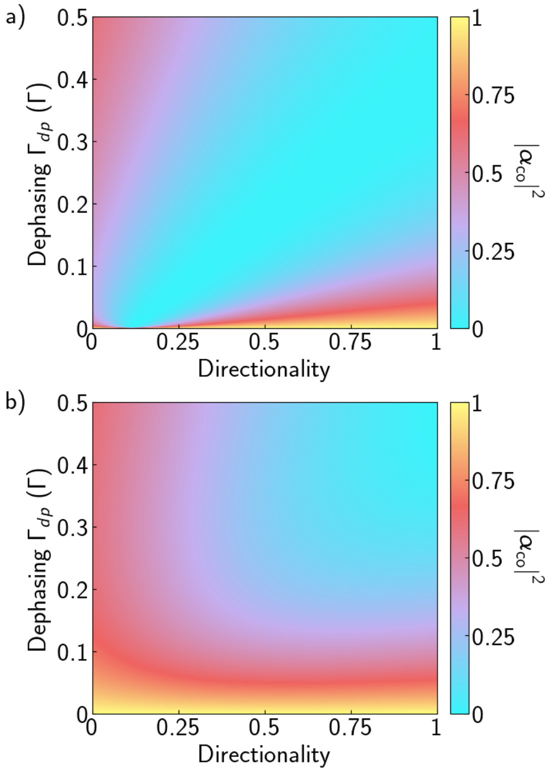

Fig. S2: Coherent phase shift probability ($|\alpha_{\mathrm{co}}|^2$) maps spanning dephasing (0Γ to 0.5Γ) and directionality (0 to 1) with coupling of $\beta = 0.9$ (a) $\Delta_{\mathrm{P}} = 0\Gamma$ (b) $\Delta_{\mathrm{P}} = 0.3\Gamma$.

The detuning shift is sampled from the normal distribution shown in Figure S3a, with Figure S3b showing the phase shift distribution for phase shifts of π and $\pi/2$, and Figure S3c showing a sample MZI pre and post spectral diffusion shift. It is evident that large phase shifts (like π) where $\Delta_P \to 0$ will have large fluctuations since detuning will often change signs, whereas smaller phase shifts with larger detuning such as $\pi/2$ will not see as much spectral diffusion influence.

S3.1 Selecting Experimental Parameters for Imperfections

In Tab. S1, we provide an alternate version of Tab. 1 from the main text that includes further details on the selected typical and state-of-the-art experimental QD parameters used in the simulations. Though the uncertainties are estimated with different methods depending on the particular reference, they are included where possible to more accurately reflect the results reported in the literature. Also, we explicitly note the types of waveguides, if applicable, used in the experiments that measured these results. Here, it is evident that some of the parameters correspond to different waveguide types. As a result, it should be noted that this study was meant to remain as platform-independent as possible to provide a general overview of the proposed QD-based qPICs.

With regards to the state-of-the-art coupling, β, though Ref. [S13] reports 0.99 ± 0.01, we chose the result of Ref. [S14] due to the reduced uncertainty.

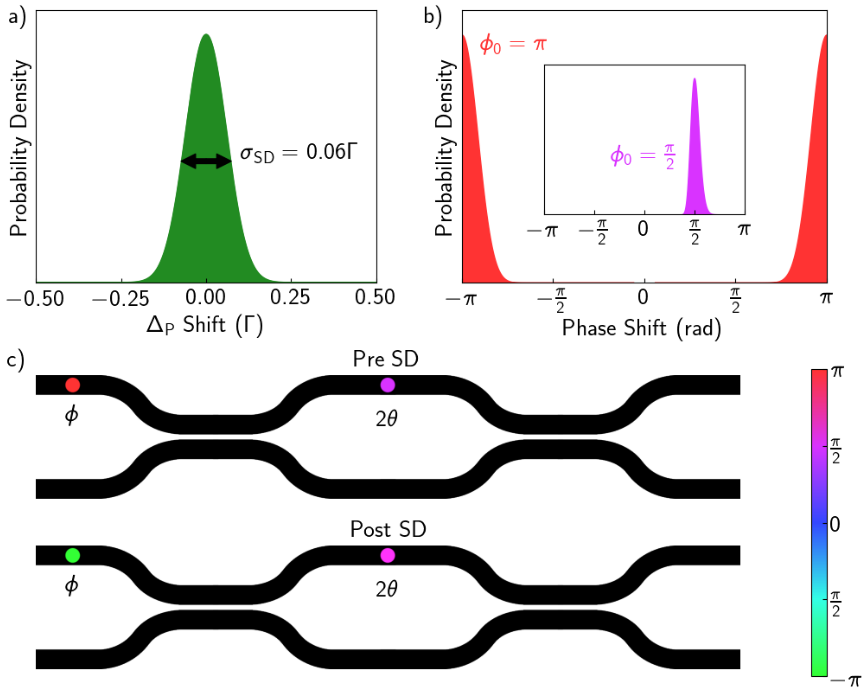

Fig. S3: Spectral Diffusion effects on detuning and phase shift for $\sigma_{SD} = 0.06\Gamma$. (a) Normal distribution for detuning shift from spectral diffusion. (b) Phase shift distribution example for initial phases of π and $\pi/2$ (inset). (c) Example MZI with initial phases of $\phi = \pi$ and $2\theta = \pi/2$ before and after sampling spectral diffusion.

Tab. S1: State-of-the-art and typical quantum dot parameters. Listed parameters include coupling, β, directionality, D, dephasing, Γ_{dp}, spectral diffusion detuning standard deviation, σ_{sd}. Uncertainties and waveguide types are reported where available and applicable (NBW: nanobeam waveguide, PhC: photonic crystal waveguide, GPW: glide-plane photonic crystal waveguide, N/A: no waveguide). When there are multiple references, the waveguide types are listed in order.

	Typical	Waveguide	Ref(s)	State-of-the-art	Waveguide	Ref(s)
β	0.90	GPW	[S15]	0.9843 ± 0.0004	PhC	[S14]
D	0.90 ± 0.013	GPW	[S15]	0.95 ± 0.05	NBW	[S16]
Γ_{dp}	0.01Γ	PhC	[S17]	0Γ	PhC, GPW, N/A, N/A, N/A	[S15, S17–S20]
σ_{sd}	0.06Γ	NBW	[S21]	0Γ	PhC, N/A	[S22, S23]

S4 Circuit Accuracy Measurement Methods and Phase Optimization

The accuracy of an imperfect transfer matrix (non-ideal U) for each circuit is calculated using the matrix infidelity [S6],

$$\mathcal{I} = 1 - \left| \frac{\text{tr}(\text{U}^{\dagger}\text{U}_{\text{non}})}{\sqrt{N\text{tr}(\text{U}^{\dagger}_{\text{non}}\text{U}_{\text{non}})}} \right|^2, \tag{S29}$$

which excludes balanced losses, allowing us to focus on accuracy over count rate. However, for the CZ and CNOT gate, we consider accuracy using the post-selected output state infidelity instead. Since the CNOT and CZ are two-photon gates, their matrices must be converted into the fock basis by calculating the matrix permanent [S24]. However, only four inputs/outputs of these expanded matrices correspond to the computational two-qubit basis states $\{|00\rangle, |01\rangle, |10\rangle, |11\rangle\}$. Thus, for each

input-output pair, we calculate the unconditional output state following the equation,

$$|\psi_{\text{out,non}}^{(i,\text{unc})}\rangle = U_{\text{non}}|\psi_{\text{in}}^{(i)}\rangle, \tag{S30}$$

where $|\psi_{\text{out,non}}^{(i,\text{unc})}\rangle$ is the imperfect unconditional output state in the fock basis, and U_{non}, $\psi_{\text{in}}^{(i)}$ are also in the fock basis. These states are then post-selected by truncating them down to the computational basis states resulting in four-entry vectors and re-normalizing them. The conditional output state infidelity for an input-output pair is then calculated using,

$$\mathcal{I}_i^{(\text{con})} = 1 - \left|\left\langle\psi_{\text{out}}^{(i,\text{con})}|\psi_{\text{out,non}}^{(i,\text{con})}\right\rangle\right|^2. \tag{S31}$$

We also consider the post-selected 4×4 computational basis matrices in the main text, which are found by truncating the fock basis matrix down to the 4×4 matrix corresponding to the four computational basis inputs and outputs and re-normalizing the matrix.

To perform phase shift optimization on these circuits, we use the appropriate cost function (Equation S29 or S31) to measure the circuit error, and perform an optimization on all phases using the BOBYQA optimization algorithm [S25]. To do this, we began by determining the phase shifts for a given unitary in the ideal case using the Clements decomposition method outlined in Section S2. Then, imperfections were added to the circuit to calculate the imperfect transfer matrix and its associated infidelity. For optimization, the ideal phases were chosen as initial phase guesses, with phase constraints of $[-\pi, \pi]$, which are required as this is a constrained optimization algorithm. When optimizing circuits with dephasing/spectral diffusion, the cost function averages the infidelity for 500 sampled matrices to find the cost for every optimization step.

S5 CNOT Gate Results

Similar to the results for the CZ gate in the main text, here we consider the unheralded CNOT gate, a 6-mode circuit with 1/9 probability of success. The performance was first measured using the matrix infidelity (Equation S29) spanning from dephasing of $\Gamma_{dp} = 10^{-8}\Gamma \rightarrow 10^{-1}\Gamma$ for nanophotonic, state-of-the-art and typical imperfections as shown in Figure S4. These imperfections are the same as listed in the main text. The results show that performance and optimization depend heavily on dephasing, where incoherent interactions heavily hinder performance. However, state-of-the-art quantum dot imperfections can be optimized to perform on par with nanophotonic imperfections with no dephasing. Next, as described in Section S4, the CNOT gate circuit was optimized based on the average conditional output state infidelity (Equation S31). The optimized post-selected 4×4 computational basis matrices for nanophotonic, state-of-the-art, and typical imperfections are shown in Figure S4b-d, along with their optimized conditional output state infidelities. The output state performance is near perfect for nanophotonic and state-of-the-art imperfections, despite overall performing poorer than the CZ due to increased circuit complexity.

S6 Random Circuit Infidelity Distributions

Each infidelity data point in the results is the result of a beta distribution across 100 Haar random unitary matrices to simulate random circuit performance accurately. A beta distribution is used to accurately average the data as the infidelity distribution across 100 samples is asymmetric and includes outliers. A beta distribution follows the probability density function,

$$f(x) = \frac{(x-a)^{p-1}(b-x)^{q-1}}{B(p,q)(b-a)^{p+q-1}}, \tag{S32}$$

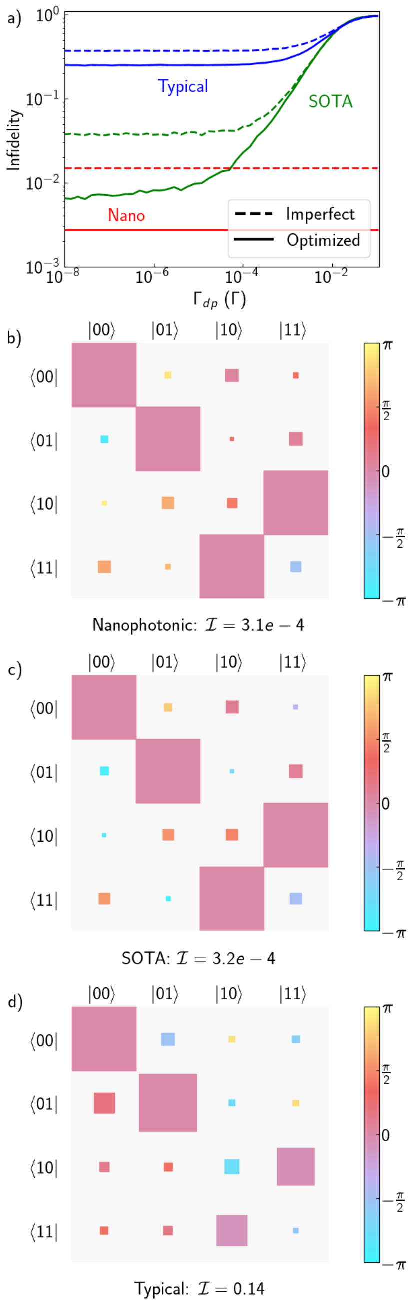

Fig. S4: Unheralded CNOT performance for nanophotonic, state-of-the-art, and typical imperfections. (a) Matrix infidelity as a function of dephasing. The nanophotonic infidelities with no dephasing are shown as horizontal lines across the figure. (b-d) Optimized nanophotonic, state-of-the-art, and typical post-selected 4×4 computational basis matrices for the unheralded CNOT gate, with conditional output state fidelities listed.

where a and b are the lower and upper bounds on x, p and q are shape parameters where $p, q > 0$ and $B(p, q)$ is the beta function that follows the equation,

$$B(\alpha, \beta) = \int_0^1 t^{\alpha-1}(1-t)^{\beta-1}dt. \tag{S33}$$

Figure S5 shows an example infidelity histogram for an $N = 4$ circuit with an arbitrary choice of 2.3% beam splitter error. This figure also plots a beta fit, normal distribution fit, and the mean of the data. The mean and normal fit in Figure S5 both result in average infidelity that is higher than

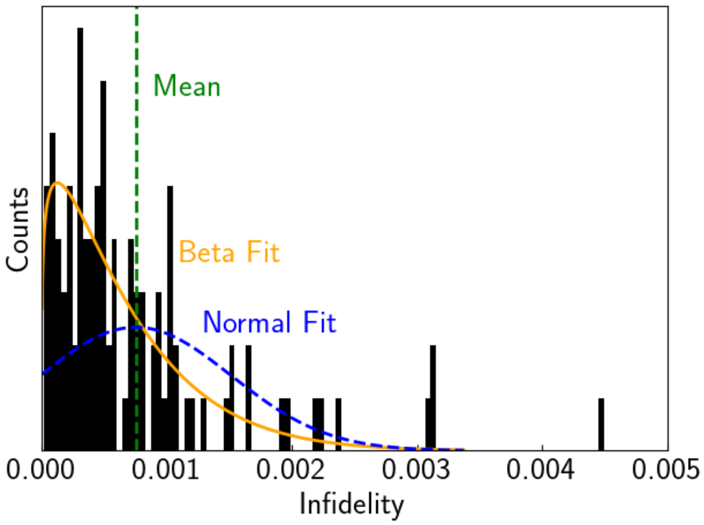

Fig. S5: Matrix infidelity histogram over 100 Haar random $N = 4$ circuits for a beam splitter error of 2.3%, with no other imperfections. Here we show the mean of the data (green), a beta fit (orange), and a normal fit (blue), to the data.

the majority of the data. Conversely, the beta fit accounts for the asymmetry, representing the data accurately. Thus, the mean of the beta fit is used for random circuit infidelity results.

S7 Linear Response Pulse Width Constraint

The scattering of few-photon Fock states by a two-level system chirally-coupled to a one-dimensional waveguide can be described using an input-output formalism, as demonstrated in [S26]. Here, we will use the scattering matrices for one and two-photon Fock states to demonstrate the linear phase response that arises when the photon pulse width σ_p is much less than the linewidth of the quantum emitter Γ. Then, we select a practical constraint for σ_p where the emitter can be used as a linear phase shifter for Fock states of up to two photons. For simplicity, we will assume perfect directionality and no loss.

As shown in [S26], the single-photon scattering matrix elements are given by,

$$\langle p| S |q\rangle = t(q)\delta(q-p) \quad : \quad t(q) \equiv \frac{q - \omega_A - \frac{i\Gamma}{2}}{q - \omega_A + \frac{i\Gamma}{2}}, \tag{S34}$$

where S is the scattering matrix, $|q\rangle = a_q^\dagger |0\rangle$, and ω_A is the transition frequency of the quantum emitter. Consider a single-photon wave packet with a pulse shape $\alpha(\omega)$, centered at angular frequency

ω_0, as the input state,

$$|\text{in}\rangle_1 = \int_{-\infty}^{\infty} d\omega \alpha(\omega) a_\omega^\dagger |0\rangle . \tag{S35}$$

The output state can be derived as,

$$|\text{out}\rangle_1 = \int_{-\infty}^{\infty} d\omega t(\omega)\alpha(\omega) a_\omega^\dagger |0\rangle , \tag{S36}$$

by first applying the scattering matrix to the input, then inserting closure followed by Eq. S34. Most commonly, $\alpha(\omega)$ will take either a Gaussian or Lorentzian form, both of which can be parameterized by width σ_p. For either form, $\alpha(\omega) \sim \delta(\omega - \omega_0)$ in the limit $\sigma_\text{p} \to 0$ which implies,

$$|\text{out}\rangle_1 \sim t(\omega_0) a_{\omega_0}^\dagger |0\rangle . \tag{S37}$$

In the lossless case, $|t(\omega)| = 1$ and $\arg\{t(\omega)\}$ varies with detuning $\Delta = \omega_A - \omega_0$ from $-\pi$ to π. Therefore, the quantum emitter acts as a perfect phase shifter when acting on single-photon Fock states in the monochromatic limit.

We now turn to the two-photon scattering matrix elements, derived in Ref. [S26] as,

$$\begin{aligned} \langle p_1 p_2 | S | q_1 q_2 \rangle = \;& t(p_1)t(p_2)\left[\delta(q_1 - p_1)\delta(q_2 - p_2) + \delta(q_1 - p_2)\delta(q_2 - p_1)\right] \\ & + \frac{i\sqrt{\Gamma}}{\pi} s(p_1)s(p_2)\left[s(q_1) + s(q_2)\right]\delta(q_1 + q_2 - p_1 - p_2), \end{aligned} \tag{S38}$$

where $|q_1 q_2\rangle = \frac{1}{\sqrt{2}} a_{q1}^\dagger a_{q2}^\dagger |0\rangle$ and $s(\omega)$ is a measure of the excitation of the emitter by a single-photon wave packet, defined by,

$$s(\omega) \equiv \frac{\sqrt{\Gamma}}{q - \omega_A + \frac{i\Gamma}{2}}. \tag{S39}$$

With the input as the two-photon analog of Eq. S35,

$$|\text{in}\rangle_2 = \frac{1}{\sqrt{2}} \int_{-\infty}^{\infty} d\omega_1 \int_{-\infty}^{\infty} d\omega_2 \alpha(\omega_1)\alpha(\omega_2) a_{\omega_1}^\dagger a_{\omega_2}^\dagger |0\rangle , \tag{S40}$$

the output can be derived by following the same procedure to obtain the result,

$$\begin{aligned} |\text{out}\rangle_2 = & \frac{1}{\sqrt{2}} \int_{-\infty}^{\infty} d\omega_1 \int_{-\infty}^{\infty} d\omega_2 \Bigg[t(\omega_1)t(\omega_2)\alpha(\omega_1)\alpha(\omega_2) + \frac{i\sqrt{\Gamma}}{2\pi} s(\omega_1)s(\omega_2) \\ & \times \int_{-\infty}^{\infty} dp \alpha(\omega_1 + \omega_2 - p)\alpha(p)\left(s(\omega_1 + \omega_2 - p) + s(p)\right) \Bigg] a_{\omega_1}^\dagger a_{\omega_2}^\dagger |0\rangle . \end{aligned} \tag{S41}$$

This output state consists of the sum of a purely uncorrelated part with a part that features correlations between the photons, however, these parts are not orthogonal. Therefore, it is not trivial to show analytically that only a linear phase response occurs in the limit $\sigma_p \to 0$. Instead, given that the input state is purely uncorrelated, we plot the input-output overlaps in Fig. S6 for one and two-photon Fock states as a function of σ_p assuming a Lorentzian pulse shape,

$$\alpha(\omega) = \sqrt{\frac{2}{\pi}} \frac{\sqrt{\sigma_\text{p}^3}}{\sigma_\text{p}^2 + (\omega - \omega_0)^2}, \tag{S42}$$

for convenience, yet without loss of generality in the limit $\sigma_\text{p} \to 0$, and resonance such that $\omega_A = \omega_0$. We find that these overlaps are purely real for all σ_p, and are able to clearly identify the desired linear phase response,

$$|\text{out}\rangle_n = e^{i \arg\{t(\omega_0)\}} |\text{in}\rangle_n , \tag{S43}$$

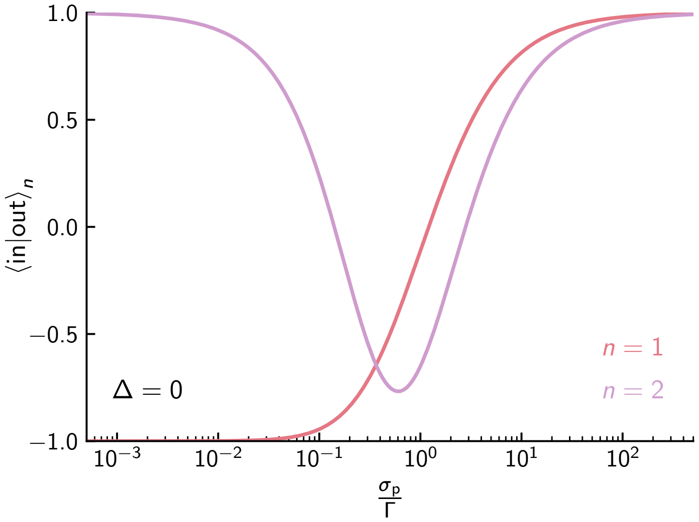

Fig. S6: Overlap between an input uncorrelated n-photon wave packet with Lorentzian pulse shape and the output state achieved after the photons scatter from a perfectly chiral two-level quantum emitter coupled to a 1D waveguide, as a function of pulse width σ_p. Each photon is assumed to be centered on resonance with the quantum emitter transition frequency such that $\Delta = \omega_A - \omega_0 = 0$, and the interaction is assumed to be lossless.

for $\sigma_\mathrm{p} \leq 0.001\Gamma$, where $\arg\{t(\omega_0)\} = \pi$ on resonance. Specifically, the magnitudes of the input-output overlaps are both > 0.99 if this constraint is met.

In other words, the condition we have derived states that the photons input to a QD phase shifter should be approximately monochromatic relative to the QD to avoid reshaping. This implies that the linewidth of the QD is a degree-of-freedom which may be tuned by engineering the surrounding nanophotonic environment to achieve this requirement. For example, if the QD is placed in a cavity, its linewidth may be broadened by enhancing the emission. In contrast, subradiant states of coherently-coupled quantum emitters may be used to slow emission.

References

[S1] C. Gerry and P. L. Knight, *Introductory quantum optics*. Cambridge university press, 2005.

[S2] P. Meystre and M. Sargent, *Elements of quantum optics*. Springer Berlin Heidelberg, 2007.

[S3] A. Asenjo-Garcia, J. Hood, D. Chang, and H. Kimble, "Atom-light interactions in quasi-one-dimensional nanostructures: A green's-function perspective," *Physical Review A*, vol. 95, no. 3, p. 033818, 2017.

[S4] H. T. Dung, L. Knöll, and D.-G. Welsch, "Resonant dipole-dipole interaction in the presence of dispersing and absorbing surroundings," *Physical Review A*, vol. 66, no. 6, p. 063810, 2002.

[S5] D. Dzsotjan, J. Kästel, and M. Fleischhauer, "Dipole-dipole shift of quantum emitters coupled to surface plasmons of a nanowire," *Physical Review B*, vol. 84, no. 7, p. 075419, 2011.

[S6] W. R. Clements, P. C. Humphreys, B. J. Metcalf, W. S. Kolthammer, and I. A. Walmsley, "Optimal design for universal multiport interferometers," *Optica*, vol. 3, no. 12, pp. 1460–1465, 2016.

[S7] W. Bogaerts, D. Pérez, J. Capmany, D. A. B. Miller, J. Poon, D. Englund, F. Morichetti, and A. Melloni, "Programmable photonic circuits," *Nature*, vol. 586, no. 7828, pp. 207–216, Oct. 2020. [Online]. Available: https://www.nature.com/articles/s41586-020-2764-0

[S8] J. Ewaniuk, J. Carolan, B. J. Shastri, and N. Rotenberg, "Imperfect Quantum Photonic Neural Networks," *Advanced Quantum Technologies*, vol. 6, no. 3, p. 2200125, Mar. 2023. [Online]. Available: https://onlinelibrary.wiley.com/doi/10.1002/qute.202200125

[S9] F. Shokraneh, S. Geoffroy-gagnon, and O. Liboiron-Ladouceur, "The diamond mesh, a phase-error- and loss-tolerant field-programmable mzi-based optical processor for optical neural networks," *Opt. Express*, vol. 28, no. 16, pp. 23 495–23 508, Aug 2020. [Online]. Available: https://opg.optica.org/oe/abstract.cfm?URI=oe-28-16-23495

[S10] F. Shokraneh, M. Sanadgol Nezami, and O. Liboiron-Ladouceur, "Theoretical and Experimental Analysis of a 44 Recongurable MZI-Based Linear Optical Processor," *Journal of Lightwave Technology*, pp. 1–1, 2021. [Online]. Available: https://ieeexplore.ieee.org/document/9383831/

[S11] S. Bandyopadhyay, R. Hamerly, and D. Englund, "Hardware error correction for programmable photonics," *Optica*, vol. 8, no. 10, pp. 1247–1255, Oct 2021. [Online]. Available: https://opg.optica.org/optica/abstract.cfm?URI=optica-8-10-1247

[S12] A. Ribeiro, A. Ruocco, L. Vanacker, and W. Bogaerts, "Demonstration of a 4 × 4-port universal linear circuit," *Optica*, vol. 3, no. 12, pp. 1348–1357, Dec 2016. [Online]. Available: https://opg.optica.org/optica/abstract.cfm?URI=optica-3-12-1348

[S13] L. Scarpelli, B. Lang, F. Masia, D. M. Beggs, E. A. Muljarov, A. B. Young, R. Oulton, M. Kamp, S. Höfling, C. Schneider, and W. Langbein, "99% beta factor and directional coupling of quantum dots to fast light in photonic crystal waveguides determined by spectral imaging," *Phys. Rev. B*, vol. 100, p. 035311, Jul 2019. [Online]. Available: https://link.aps.org/doi/10.1103/PhysRevB.100.035311

[S14] M. Arcari, I. Söllner, A. Javadi, S. Lindskov Hansen, S. Mahmoodian, J. Liu, H. Thyrrestrup, E. H. Lee, J. D. Song, S. Stobbe, and P. Lodahl, "Near-unity coupling efficiency of a quantum emitter to a photonic crystal waveguide," *Phys. Rev. Lett.*, vol. 113, p. 093603, Aug 2014. [Online]. Available: https://link.aps.org/doi/10.1103/PhysRevLett.113.093603

[S15] I. Söllner, S. Mahmoodian, S. L. Hansen, L. Midolo, A. Javadi, G. Kiršanskė, T. Pregnolato, H. El-Ella, E. H. Lee, J. D. Song, S. Stobbe, and P. Lodahl, "Deterministic photon–emitter coupling in chiral photonic circuits," *Nature Nanotechnology*, vol. 10, no. 9, pp. 775–778, Sep. 2015. [Online]. Available: https://www.nature.com/articles/nnano.2015.159

[S16] R. J. Coles, D. M. Price, J. E. Dixon, B. Royall, E. Clarke, P. Kok, M. S. Skolnick, A. M. Fox, and M. N. Makhonin, "Chirality of nanophotonic waveguide with embedded quantum emitter for unidirectional spin transfer," *Nature Communications*, vol. 7, no. 1, p. 11183, Mar. 2016. [Online]. Available: https://www.nature.com/articles/ncomms11183

[S17] H. Le Jeannic, A. Tiranov, J. Carolan, T. Ramos, Y. Wang, M. H. Appel, S. Scholz, A. D. Wieck, A. Ludwig, N. Rotenberg *et al.*, "Dynamical photon–photon interaction mediated by a quantum emitter," *Nature Physics*, vol. 18, no. 10, pp. 1191–1195, 2022.

[S18] W. Langbein, P. Borri, U. Woggon, V. Stavarache, D. Reuter, and A. Wieck, "Radiatively limited dephasing in inas quantum dots," *Physical Review B*, vol. 70, no. 3, p. 033301, 2004.

[S19] C. Matthiesen, A. N. Vamivakas, and M. Atatüre, "Subnatural linewidth single photons from a quantum dot," *Physical Review Letters*, vol. 108, no. 9, p. 093602, 2012.

[S20] H.-S. Nguyen, G. Sallen, C. Voisin, P. Roussignol, C. Diederichs, and G. Cassabois, "Ultra-coherent single photon source," *Applied Physics Letters*, vol. 99, no. 26, 2011.

[S21] H. Thyrrestrup, G. Kiršanskė, H. Le Jeannic, T. Pregnolato, L. Zhai, L. Raahauge, L. Midolo, N. Rotenberg, A. Javadi, R. Schott, A. D. Wieck, A. Ludwig, M. C. Löbl, I. Söllner, R. J. Warburton, and P. Lodahl, "Quantum Optics with Near-Lifetime-Limited Quantum-Dot Transitions in a Nanophotonic Waveguide," *Nano Letters*, vol. 18, no. 3, pp. 1801–1806, Mar. 2018. [Online]. Available: https://doi.org/10.1021/acs.nanolett.7b05016

[S22] F. T. Pedersen, Y. Wang, C. T. Olesen, S. Scholz, A. D. Wieck, A. Ludwig, M. C. Löbl, R. J. Warburton, L. Midolo, R. Uppu *et al.*, "Near transform-limited quantum dot linewidths in a broadband photonic crystal waveguide," *ACS Photonics*, vol. 7, no. 9, pp. 2343–2349, 2020.

[S23] A. V. Kuhlmann, J. Houel, A. Ludwig, L. Greuter, D. Reuter, A. D. Wieck, M. Poggio, and R. J. Warburton, "Charge noise and spin noise in a semiconductor quantum device," *Nature Physics*, vol. 9, no. 9, pp. 570–575, 2013.

[S24] S. Aaronson and A. Arkhipov, "The computational complexity of linear optics," 2010.

[S25] M. J. Powell *et al.*, "The BOBYQA algorithm for bound constrained optimization without derivatives," *Cambridge NA Report NA2009/06, University of Cambridge, Cambridge*, vol. 26, 2009.

[S26] S. Fan, Ş. E. Kocabaş, and J.-T. Shen, "Input-output formalism for few-photon transport in one-dimensional nanophotonic waveguides coupled to a qubit," *Physical Review A*, vol. 82, no. 6, p. 063821, 2010.
